# Effects of oncological care pathways in primary and secondary care on patient, professional, and health systems outcomes: protocol for a systematic review and meta-analysis

**DOI:** 10.1186/s13643-018-0693-x

**Published:** 2018-03-27

**Authors:** Jolanda C. van Hoeve, Robin W. M. Vernooij, Adegboyega K. Lawal, Michelle Fiander, Peter Nieboer, Sabine Siesling, Thomas Rotter

**Affiliations:** 1 Department Health Technology and Services Research (HTSR), University of Twente, P.O. Box 217, 7500 AE Enschede, the Netherlands; 2 Netherlands Comprehensive Cancer Organisation (IKNL), P.O. Box 19079, 3501 DB Utrecht, the Netherlands; 3 College of Pharmacy and Nutrition, University of Saskatchewan, Saskatoon, Canada; 4 College of Pharmacy, Department of Pharmacology, University of Utah, Salt Lake City, USA; 5 Wilhelmina Hospital, Assen, the Netherlands; 6 Healthcare Quality Programs, School of Nursing, Queen’s University, Kingston, Ontario Canada

**Keywords:** Care pathways, Clinical pathways, Integrated care pathways, Care maps, Oncology, Cancer, Systematic reviews

## Abstract

**Background:**

The high impact of a cancer diagnosis on patients and their families and the increasing costs of cancer treatment call for optimal and efficient oncological care. To improve the quality of care and to minimize healthcare costs and its economic burden, many healthcare organizations introduce care pathways to improve efficiency across the continuum of cancer care. However, there is limited research on the effects of cancer care pathways in different settings.

**Methods:**

The aim of this systematic review and meta-analysis described in this protocol is to synthesize existing literature on the effects of oncological care pathways. We will conduct a systematic search strategy to identify all relevant literature in several biomedical databases, including Cochrane library, MEDLINE, Embase, and CINAHL. We will follow the methodology of Cochrane Effective Practice and Organisation of Care (EPOC), and we will include randomized trials, non-randomized trials, controlled before-after studies, and interrupted time series studies. In addition, we will include full economic evaluations (cost-effectiveness analyses, cost-utility analyses, and cost-benefit analyses), cost analyses, and comparative resource utilization studies, if available. Two reviewers will independently screen all studies and evaluate those included for risk of bias. From these studies, we will extract data regarding patient, professional, and health systems outcomes. Our systematic review will follow the PRISMA set of items for reporting in systematic reviews and meta-analyses.

**Discussion:**

Following the protocol outlined in this article, we aim to identify, assess, and synthesize all available evidence in order to provide an evidence base on the effects of oncological care pathways as reported in the literature.

**Systematic review registration:**

PROSPERO CRD42017057592.

**Electronic supplementary material:**

The online version of this article (10.1186/s13643-018-0693-x) contains supplementary material, which is available to authorized users.

## Background

### Description of the condition

Cancer (malignant neoplasm) is a generic term for a large group of diseases that can affect any part of the body. One of its defining features is the rapid growth of abnormal cells beyond their usual boundaries that can then invade adjoining parts of the body and/or spread to other organs; this process is referred to as metastasis formation and is the major cause of death from cancer [1].

As one of the leading causes of morbidity and mortality worldwide, cancer is a major public issue. Data from 2012 showed 14 million new cases and 8.8 million cancer-related deaths worldwide [2]. The most commonly diagnosed cancers were lung (1.82 million), breast (1.67 million), and colorectal (1.36 million). Similarly, the most common causes of cancer death were lung cancer (1.6 million deaths), liver cancer (745,000 deaths), and stomach cancer (723,000 deaths). The incidence data were derived from population-based cancer registries: however, these cancer registries do not cover all national populations [2]. Advances in medical technology have contributed to the growth of an aging population with a greater life expectancy but will at the same time lead to an ever-increasing cancer burden over the next decades, particularly in low- and middle-income countries, where over 20 million new cancer cases are expected annually by 2025 [3].

Given the global incidence of cancer and its mortality, cancer imposes a substantial economic burden on society. Considerable healthcare costs are associated with its prevention and management, and indirect costs result from patients’ inability to work [4]. Warren et al. state that the costs of initial cancer treatments are steadily increasing, as more patients are receiving surgery and adjuvant therapy, and the costs of these treatments are rising [5].

To minimize the burden of cancer for patients, healthcare professionals, and health care organizations, effective and efficient organization of cancer care is vital. Cancer care is complex and depends upon careful coordination between various healthcare organizations and providers. Regular communication and exchange of technical information between all those involved in treatment (including patients, general practitioners, medical specialists, and other specialty disciplines) must be guaranteed [6]. One way to provide patient-centered care, reduce waiting times, and improve the quality of cancer care is to introduce care pathways [7, 8].

### Description of the intervention

Care pathways, also known as “integrated care pathways,” “clinical pathways,” “critical pathways,” or “care plans,” have been implemented since the 1980s as tools to facilitate evidence-based healthcare. Kinsman et al. developed five criteria to define a care pathway [9]. Rotter et al. tested these criteria and refined the definition into four criteria, including a structured multidisciplinary plan of care, which translates guidelines into local structures, detailing the steps in a course of treatment or care in a plan of actions, and aiming to standardize care [10].

From the perspective of healthcare professionals and organizations, care pathways are emerging as welcome strategies for quality improvement. They help to improve multidisciplinary communication and care planning, optimize the safety and quality of care, and increase patient satisfaction [11]. Focusing on the whole pathway of care, including the care delivered to patients from diagnosis, through treatment, to living with and beyond cancer, care pathways organize efficient transitions between healthcare organizations. They are able to link evidence to practice via integration of guidelines into local systems and, consequently, optimize patient outcomes and maximize clinical efficiency [12]. The systematic review of Rotter et al. showed that care pathways reduced in-hospital complications and improved documentation among healthcare professionals. These authors also found that most studies reported a decreased length of stay and a reduction in hospital costs after care pathways were implemented [12]. However, in spite of some evidence for the use and effects of oncological care pathways, this evidence is often limited to only a small number of pathways or only a limited part of a pathway; available studies report the effects of care pathways for breast cancer [13–16], colorectal cancer [17], lung cancer [18], and prostate cancer [19].

Evidence is lacking not only about the use and effects of care pathways but also about the implementation of these pathways. Studies of strategies for implementation of guidelines, rather than pathways, are more common [20, 21]. Furthermore, Jabbour et al. stated that the true impact of pathways has been limited because the implementation strategies are variable and the research designs are suboptimal [22]. Therefore, it is important to identify the existing knowledge about implementation and the circumstances under which care pathways lead to improved care.

Although care pathways are frequently applied in cancer care, to our knowledge, systematic reviews of the whole pathway of oncology care and its effects are not available. We therefore present the systematic review as described in this protocol, for which we follow the Preferred Reporting Items for Systematic Review and Meta-Analyses (PRISMA) (see Additional file 1) [23], in order to improve the current knowledge based on the effects of oncological care pathways.

## Methods

Review questions and objectives

1. The primary question is: What are the effects of oncological care pathways on patient, professional, and health system outcomes within primary and secondary (hospital) care settings?
2. The secondary review questions are: (1) What are the differences in the implementation of oncological care pathways in primary and secondary (hospital) care? And (2) can we explain how these differences might lead to different outcomes?

For the purpose of this review, we will define usual care as treatment determined at the discretion of the attending healthcare professional. Unless studies specify that the control group utilizes some form of standardized care, we will assume the control group utilizes this definition of usual care.

### Criteria for considering studies for this review

We will include all studies that address the questions and objectives of our systematic review. We will include studies which tested the implementation of care pathways in primary and secondary (hospital) care and of intersectional care pathways in all healthcare settings.

### Types of studies

We will include all primary, quantitative studies which utilize the following study designs: randomized trials, non-randomized trials, controlled before-after studies, and interrupted time series studies according to the EPOC study design criteria [24]. Where available, we will include full economic evaluations (cost-effectiveness analyses, cost-utility analyses, and cost-benefit analyses), cost analyses, and comparative resource utilization studies. We will exclude retrospective cohort studies, prospective cohort studies, cross-sectional studies, and case-control studies.

### Definition of care pathways

Because there are many definitions of “care pathways” [9], we will use the working description which was tested and refined in a previous review by Rotter et al. [10]:

1. The intervention was a structured, multidisciplinary plan of care.
2. The intervention was used to translate guidelines or evidence into local structures.
3. The intervention detailed steps in a course of treatment or care in a plan, pathway, algorithm, guideline, protocol, or other “inventory of actions” (i.e., the intervention had time frames or criteria-based progression).
4. The intervention aimed to standardize care for a population of cancer patients.

An intervention is considered to be a care pathway only if it meets all four criteria. Otherwise, the study will be excluded.

### Types of institutions and participants

Participants will include patients, care providers, and healthcare organizations in primary and secondary care. All cancer types will be included. Regarding the care providers, we will consider all health professionals, including general practitioners, doctors, nurses, physiotherapists, pharmacists, occupational therapists, social workers, dietitians, psychologists, psychiatrists, and dentists involved in oncological care pathway utilization. Finally, we will group the included studies into primary and secondary care pathways. Also, we will synthesize and report on the studies according to the context in which they have been implemented (primary or secondary care).

### Primary outcomes

Objectively measured patient outcomes include (in-patient) mortality, mortality at the end of follow-up, re-admissions (hospital setting), (in-hospital) complications, hospital admissions, adverse events, discharge destinations, performance status, patient satisfaction, quality of life, and absence from work.

Objectively measured professional outcomes include quality measures appropriate to the specific aim of the care pathway, staff satisfaction, team functioning, guideline adherence, and adherence to evidence-based practice.

Objectively defined systems-level outcomes include length of stay, waiting times, and costs.

### Secondary outcomes

Any reported measure of the following implementation strategies and methods will be included. We will use the evidence-informed strategies identified and employed by the Cochrane authors in their systematic review, although Rotter et al. [12] found that reporting of implementation processes was generally poor. Further, we will extract the reported evidence-based processes for developing and implementing care pathways in primary and secondary care. We will group the reported implementation activities according to whether they used evidence-informed strategies. We will categorize reported strategies into (1) pathway development [12, 25], (2) implementation planning [12, 26], (3) pathway education [12, 25, 27], and (4) feedback and reminder systems [12, 27, 28].

Potential activities involved in pathway implementation include project groups, clinician involvement, local consensus processes, use of an implementation team, identification of potential barriers to change, identification of evidence-practice gaps, local opinion leaders, educational meetings and outreach, printed educational materials, audit and feedback, and electronic reminder systems.

### Search methods

We will search the Cochrane Database of Systematic Reviews and the Database of Abstracts of Reviews of Effects (DARE) to identify related systematic reviews. The initial search strategy has been developed for OVID MEDLINE and will be translated for other databases (see Additional file 2). We will apply three methodological filters: randomized controlled trials, EPOC designs (controlled before-after studies, interrupted time series studies, and quasi-experimental designs), and economic studies to identify cost-effectiveness analyses, cost-utility analyses, and cost-benefit analyses. Search strategies will use controlled vocabulary such as Medical Subject Headings (MeSH) and EMTREE (Embase), and keyword phrases. All databases will be searched from the date of inception forward with neither date nor language limits.

The databases searched are the following:

- Cochrane Central Register of Controlled Trials, Cochrane Library (Wiley)
- DARE, NHS Economic Evaluation Database (EED), Health Technology Assessment Database (HTA) (Wiley)
- MEDLINE, Epub Ahead of Print; In-Process & Other Non-Indexed; MEDLINE R; MEDLINE R Daily (OVID) 1946 to present
- Embase (OVID) 1947 to present
- CINAHL (EBSCO) 1981 to present
- Latin American and Caribbean Health Science Information database (LILACS); via Virtual Health Library, http://lilacs.bvsalud.org/en/ 1982 to present
- Scientific Electronic Library Online (SciELO) http://www.scielo.org/php/index.php 1940 to present
- The American Economic Association’s database for economic literature, EconLit (EBSCO) 1969 to present

### Searching other resources

#### Trial registries

- ClinicalTrials.gov—US National Library of Medicine https://clinicaltrials.gov
- International Clinical Trials Registry Platform (ICTRP)—World Health Organization http://apps.who.int/trialsearch/

#### Gray literature

We will search a selection of gray literature sources to identify citations not indexed in the standard bibliographic databases above. Sites will include, but not be limited to, the following:

- Open Gray www.opengrey.eu/
- The Gray Literature Report, New York Academy of Medicine (1996 to present) http://www.greylit.org
- Open Clinical http://www.openclinical.org/home.html
- Organizational web sites and professional organizations related to clinical pathways and implementation.
  - ◦ European Pathway Association http://e-p-a.org

We will also:

- Screen the following individual journals (e.g., hand search):
- ◦ International Journal of Care Pathways
- ◦ International Journal of Integrated Care
- Review reference lists of all included studies, relevant systematic reviews/primary studies
- Contact authors of relevant studies/reviews to clarify reported published information and/or to seek unpublished results/data
- Contact researchers with expertise relevant to the review topic/ EPOC interventions.
- Conduct a cited reference search on those studies included in our review in Web of Science
- Contact the corresponding author to request full text in case of the full text is missing.

### Data extraction

Two reviewers will independently screen all titles and abstracts, using Covidence (www.covidence.org). A third reviewer will be consulted in case of disagreement between the two reviewers. We will further examine potentially relevant studies, using full-text copies.

Two review authors will independently extract data, directly from included studies. Where necessary, we will request additional information from the authors of primary studies. When we include systematic reviews in the analysis, we will assess the methodological quality of these reviews using the Assessing the Methodological Quality of Systematic Reviews (AMSTAR) tool [29].

When data are missing, we will not impute any values.

Areas of data extraction will include:

- Study characteristics: publication year, country, length of follow-up period, urban vs. rural location, and inclusion criteria
- Population characteristics (of patients): age, gender, number of patients, and type of cancer
- Population characteristics (of professionals): types of healthcare professionals, number of health professionals involved in development, and healthcare setting
- Intervention characteristics: evidence-based development and implementation strategy(ies) reported.
- Outcomes: patient, professional, and systems (means, averages, and other uncertainty measures)

### Risk of bias assessment

For randomized trials, non-randomized trials, controlled before-after studies, and interrupted time series studies, we will use the validated criteria suggested by the Cochrane EPOC group to assess the risk of bias in studies with control groups [24].

The review team will identify criteria for assessing randomized trials, non-randomized trials, and controlled before-after studies: sequence generation, allocation concealment, blinding of participants and personnel, blinding of outcome assessment, similarity of baseline measures, similarity of baseline characteristics, management of incomplete outcome data, selective outcome reporting, and other risks of bias.

The review team will also identify criteria assessing interrupted time series studies: intervention independent of other changes, shape of effect pre-specified, intervention unlikely to affect data collection, blinding of outcome assessment, management of incomplete data, selective outcome reporting, and other risks of bias.

We will summarize the results of the risk of bias assessment across studies and present them in a tabular format.

### Data analysis

We will undertake meta-analyses if we find more than two comparable studies which report similar outcomes, within similar contexts, and without statistical heterogeneity. To perform a meta-analysis, we will use RevMan [30] for calculating a pooled effect (if the clinical and statistical homogeneity across groups of studies is sufficient), using both fixed and random effect models to assess the robustness of the results [31].

### Data synthesis

We will report details on the number of retrieved references, obtained full-text papers, and the included and excluded articles. We will present results of meta-analyses using forest plots. We will adjust cost data for inflation and present these data in US dollars for a common price year.

### Assessment of heterogeneity

We will assess heterogeneity within the review and analyze the comparability of the results from individual studies (*I*^2^ = [(*Q* df)/*Q*] × 100%). The cutoff for substantial statistical heterogeneity will be an *I*^2^ greater than 50%. In addition to considering the quantitative measure of *I*^2^, we will perform the Cochran’s *Q* statistical test for heterogeneity [32].

### Assessment of reporting biases

In cases where we find more than 10 studies, we will assess potential reporting biases by visual inspection of counter-enhanced funnel plots [33, 34]. To test for funnel plot asymmetry, we will use the test proposed by Egger [35] or the modified version of the Egger test proposed by Harbord in case of small study effects in meta-analyses of controlled trials with binary endpoints [36].

### Subgroup analysis

We will perform a subgroup analysis of the reported primary and secondary outcomes.

We will group studies according to the following categories:

- Country(ies) where the study was carried out
- Setting(s) where the implementation of care pathways occurred
- Year of publication, to assess differences in outcomes reported over time
- Quality of studies: high versus low risk of bias
- Age of population: studies including children versus adults
- Cancer type for which the care pathway was developed and implemented

In our subgroup analyses, we will be using the quantity *I*^2^ for estimating heterogeneity, based on Cochran’s *Q* test, and both statistics will be provided in our forest plots to depict the pooled estimates.

### Sensitivity analysis

We will use sensitivity analyses to explore the robustness of the results by investigating the effects of including and excluding studies with high and unclear risks of bias from the analysis.

### Ongoing studies

We will describe identified ongoing studies, where available, detailing the primary author, research question(s), methods, and outcome measures. In addition, we will contact the authors of ongoing studies to request raw data for inclusion in our review.

## Discussion

The systematic review outlined in this protocol aims to identify, assess, and synthesize all quantitative studies on the effects of care pathways for oncological care meeting the EPOC study design criteria. As a result, the review will provide an evidence base for cancer care pathways regarding patient effects, cost-effectiveness, and implications for implementation. Disseminating the results of the systematic review will be done by publishing the systematic review in a peer-reviewed journal and presenting the results at relevant symposia.

The methodological quality of our systematic review will be reported by using the AMSTAR tool [29].

## Additional files

Additional file 1: PRISMA 2009 checklist. (PDF 55 kb)

Additional file 2: OVID MEDLINE search strategy oncology care pathways review. (PDF 23 kb)

## Abbreviations

CBA: Controlled before-after study
EPOC: Effective Practice and Organisation of Care
ITS: Interrupted time series
LOS: Length of stay
NRCTs: Non-randomized trial
RCTs: Randomized controlled trial
